# Management strategies and outcomes of pediatric unicameral bone cyst: a narrative review

**DOI:** 10.3389/fped.2026.1753597

**Published:** 2026-03-05

**Authors:** Juntao Ge, Kaixuan Tian

**Affiliations:** 1 Department of Pediatric Surgery, The First Affiliated Hospital of Shandong First Medical University & Shandong Provincial Qianfoshan Hospital, Shandong Engineering and Technology Research Center for Pediatric Drug Development, Jinan, Shandong, China; 2 Department of Pediatric Orthopedics, Hebei Medical University Third Hospital, Shijiazhuang, Hebei, China

**Keywords:** bone cyst, curettage, pathological fracture, pediatric, treatment

## Abstract

Unicameral bone cysts (UBCs) are benign, fluid-filled lesions that predominantly occur in the long bones of children and adolescents. Although histologically non-neoplastic, these cysts can weaken bone structure and predispose patients to pathological fractures. Despite extensive research, the optimal management of UBCs remains controversial. Current treatment strategies—ranging from simple observation to minimally invasive procedures and open surgery—lack standardized guidelines, and clinical decisions are often driven by lesion activity, fracture risk, and surgeon experience. This narrative review provides a comprehensive overview of the current understanding of pediatric UBCs, with particular emphasis on their etiology, natural history, and the evolution of management strategies. It summarizes major therapeutic approaches while focusing on their underlying rationale rather than technical details. By integrating current evidence and expert perspectives, this review highlights existing challenges and aims to assist clinicians in formulating individualized, evidence-based treatment plans that optimize both safety and long-term functional outcomes in affected children.

## Introduction

Unicameral bone cysts (UBCs), also referred to as simple bone cysts, are benign, fluid-filled osteolytic lesions typically arising in the metaphyseal regions of long bones. Radiographically, they appear as well-demarcated, radiolucent areas, often containing serous fluid ranging in color from yellow to brown (1). Although histologically non-neoplastic, UBCs can exhibit locally aggressive behavior, particularly in skeletally immature individuals. The estimated incidence is approximately 3 per million individuals annually, with a strong predilection for pediatric and adolescent populations—nearly 80% of cases are diagnosed before the age of 20-and a notable male predominance, with reported male-to-female ratios ranging from 2:1 to 3:1 (2). Anatomically, the lesions most commonly involve the proximal humerus (accounting for 50%–56% of cases) and the proximal femur, together comprising roughly three-quarters of all occurrences (3).

Given their potential to compromise structural integrity, UBCs are clinically significant due to the associated risk of pathological fractures, limb deformity, and growth disturbances. Surgical management remains the cornerstone of treatment, primarily involving curettage of the cyst wall followed by bone grafting. However, recurrence remains a substantial concern, with reported rates varying between 18% and 50% (1). Furthermore, the lack of standardized guidelines means clinical decisions are often driven by surgeon preference rather than high-level evidence. Therefore, there is an urgent need to synthesize the latest advancements in minimally invasive and regenerative therapies to establish more predictable treatment pathways. This narrative review aims to clarify the natural history of UBCs and evaluate the timing and indications for both conservative and surgical treatments. By synthesizing current evidence, we provide a framework to help clinicians choose the most effective, individualized approach for optimal healing and functional recovery.

## Methods

To provide a rigorous and reproducible foundation for this review, we conducted a structured literature search across the PubMed, Web of Science. The search encompassed all relevant studies published up to December 2025, utilizing Medical Subject Headings (MeSH) terms and keywords including “unicameral bone cyst,” “simple bone cyst,” “pediatric,” and “management.” These terms were interconnected using Boolean operators, such as (“unicameral bone cyst” OR “simple bone cyst”) AND “pediatric” AND (“treatment” OR “surgical intervention”).

Although this manuscript is a narrative review designed to systematically introduce the development and treatment of UBCs, we adopted a highly selective inclusion process inspired by evidence-based principles. We prioritized high-quality evidence, including randomized controlled trials, systematic reviews, and large-scale retrospective studies, to ensure the reliability of our conclusions. We evaluated the certainty of evidence by cross-referencing findings across diverse study designs, thereby addressing potential biases arising from the heterogeneity of clinical data. This approach ensures that while the review maintains a broad and cohesive narrative, the clinical recommendations are grounded in a transparent and verifiable bibliographic framework.

## Etiology

The pathogenesis of unicameral bone cysts (UBCs) remains incompletely understood, with several mechanisms proposed, including disturbances in bone development, fluid retention, altered bone remodeling, and venous obstruction (4, 5). Among these, the hypothesis of metaphyseal venous drainage impairment is particularly compelling, as UBCs commonly arise in the metaphysis. Angiographic findings have demonstrated dilated nutrient arteries and disrupted central medullary venous flow at the cyst site, with compensatory drainage through periosteal and distal metaphyseal veins (5, 6). This venous obstruction may lead to elevated intracystic pressure and accumulation of fluid enriched with proteolytic enzymes, prostaglandins, and reactive oxygen species, which in turn contribute to bone matrix degradation (7). Chigira et al. (8) further supported this mechanism by reporting intracystic pressures 2–3 mmHg higher than in adjacent normal bone, highlighting the role of venous hypertension in cyst formation. While the initiating cause of venous obstruction may differ among cases, this mechanism may represent a unifying feature in UBC development.

Beyond mechanical and vascular theories, cytogenetic abnormalities have also been implicated. Vayego et al. identified a complex clonal chromosomal rearrangement involving multiple chromosomes, including both homologs of chromosome 12, in a histologically benign UBCs-an atypical finding more often associated with malignant neoplasms (9). In contrast, Richkind et al. (10). reported a single balanced translocation, t(16;20)(p11.2;q13), as the sole cytogenetic anomaly in another case, suggesting heterogeneity in genetic alterations underlying UBCs. These findings point to potential genomic instability in at least a subset of lesions and underscore the need for further molecular investigations to elucidate the frequency and significance of such abnormalities.

### Natural history

Understanding the natural history of UBCs is crucial for guiding treatment and predicting prognosis. Despite extensive literature on therapeutic outcomes, the full natural course of these benign lesions remains inadequately characterized (11). UBCs often present with subtle symptoms, leading to delayed diagnosis, which commonly occurs only after a pathological fracture, trauma, pain, or gait disturbance. Radiographically, UBCs are categorized into phases based on their proximity to the physis: an Active Phase describes a cyst directly abutting the growth plate (<0.5 cm), correlating with rapid growth, high recurrence, and increased fracture risk; a Latent Phase signifies the cyst has migrated (>0.5 cm) from the physis, suggesting decelerated or arrested growth and lower recurrence rates; and a Healing Phase denotes visible new bone formation and cortical thickening, indicating spontaneous resolution or successful treatment (4, 5, 12). Observational studies highlight the inevitability of pathological fractures within the natural history, with one cohort reporting nine pathological fractures among 11 conservatively managed patients, though five ultimately experienced spontaneous healing (13). While all humeral fractures typically heal within 2–3 weeks and femoral fractures in 10 weeks, implying UBCs are self-limiting, the primary concern remains preventing such fractures (14). Notably, a pathological fracture does not alter the cyst's inherent natural course or tendency towards regression. Therefore, a comprehensive understanding of UBCs natural progression, including lesion location, size, and metaphyseal distance from the physis, is vital for predicting future development and optimizing therapeutic strategies.

### Conservative treatment

Understanding the natural history of UBCs is essential for determining appropriate treatment strategies. For small lesions in children that are discovered incidentally or present with minimal symptoms, conservative management is typically the preferred initial approach. The primary objectives are to maintain proper limb alignment, preserve functional activity, and prevent pathological fractures (15). Previous studies have demonstrated that many pediatric UBCs may spontaneously regress during skeletal maturation, underscoring the need for regular follow-up and serial imaging to monitor cyst evolution (16). According to a survey of EPOS and POSNA members, the consensus among experts is to manage asymptomatic, low-risk UBCs found by chance with active surveillance (no intervention but regular follow-up) and a precaution of restricting sports activities (17). Common conservative measures include observation, physiotherapy, and symptomatic medication. The observation period generally ranges from 6 to 12 months, during which radiographs or MRI are performed to assess changes in cyst size and morphology. If the cyst remains stable in size and the patient is asymptomatic, continued observation is appropriate. For children with mild pain or functional limitation, nonsteroidal anti-inflammatory drugs (NSAIDs) may be administered for symptomatic relief (16).

**Figure 1:**
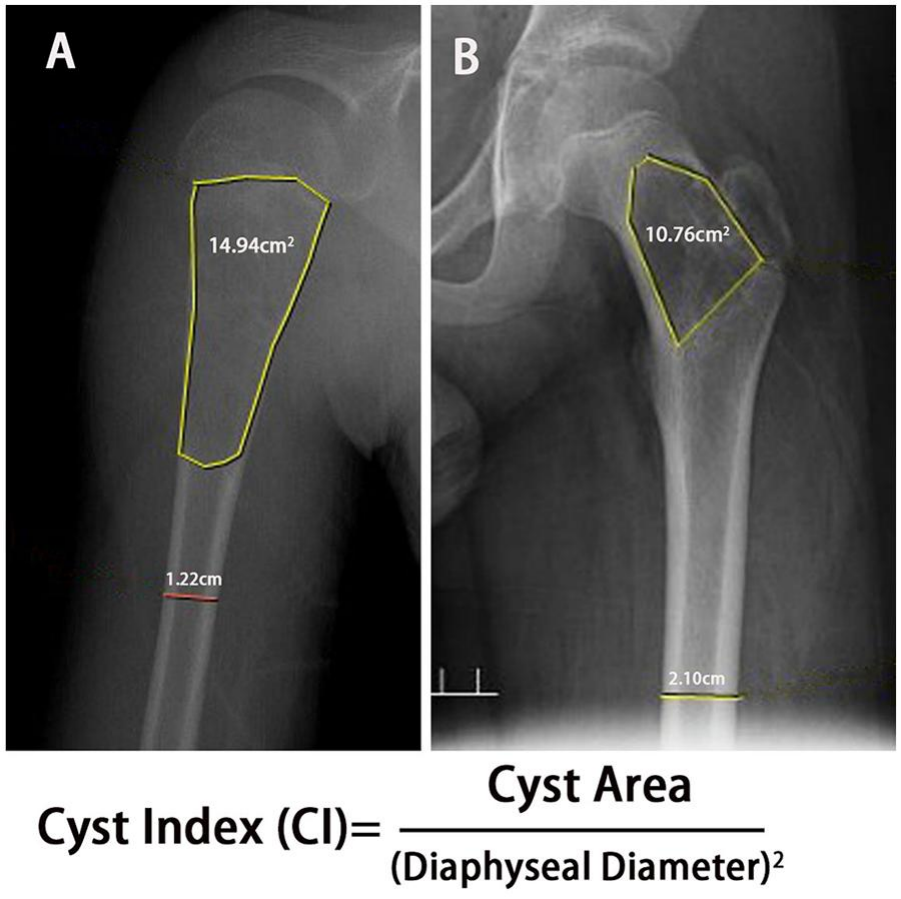
**(A, B)** Cyst index in proximal humerus and femur.

In a cohort study involving 50 pediatric patients with SBCs, Deventer et al. (15) reported that only three cases achieved spontaneous healing under conservative treatment, including those complicated by pathological fractures. This finding suggests that nonoperative management alone may have limited curative potential. Lin et al. (18) indicates a high failure rate of conservative management for pediatric pathologic proximal femur fractures, with 88% of patients initially treated with casting requiring revision surgery, leading to the recommendation for internal fixation. To objectively evaluate the structural integrity of the affected bone, Kaelin (13) introduced the concept of the Cyst Index (CI), which quantitatively relates cyst size to bone diameter. The CI is calculated by dividing the cyst area—measured on anteroposterior radiographs-by the square of the diaphyseal diameter of the affected bone at the same level. A higher CI corresponds to an increased risk of pathological fracture. According to Kaelin's data, the threshold values for initial pathological fractures were approximately 4.0 for the proximal humerus and 3.5 for the proximal femur (Figure 1). In summary, treatment selection for bone cysts should be individualized based on both fracture risk and cyst activity. Patients with a high Cyst Index or active lesions should be considered for interventional procedures, such as percutaneous injections or surgical stabilization, whereas those with low fracture risk and quiescent cysts can be safely managed with observation and regular radiological follow-up.

1. Pediatric UBCs located in the proximal humerus. The cyst area measures 14.94 cm^2^ and the diaphyseal diameter is 1.22 cm, resulting in a Cyst Index (CI) of 10. This value categorizes the lesion as High Risk (>4.0).
2. Pediatric UBCs located in the proximal femur. The cyst area measures 10.76 cm^2^ and the diaphyseal diameter is 2.1 cm, resulting in a Cyst Index (CI) of 2.43. This value categorizes the lesion as Low Risk (<3.5).

### Intralesional injections therapy

Intralesional injection serves as a fundamental minimally invasive approach in the management of UBCs. This technique involves the percutaneous delivery of therapeutic agents directly into the cyst cavity, with the goal of stimulating bone healing while avoiding the morbidity associated with open surgical procedures. The development of injectable treatments reflects an ongoing refinement in clinical strategy-progressing from early corticosteroid injections, to the regenerative use of autologous bone marrow combined with demineralized bone matrix, and more recently to engineered synthetic bone graft substitutes. Each class of agent operates through a distinct biological pathway to promote cyst resolution, and their continued evolution underscores a sustained effort to improve treatment outcomes, enhance bone regeneration, and increase practicality in clinical practice (Table 1) Steroid.

**Table 1:** Conservative and minimally invasive injection strategies for UBCs.

Treatment modality	Indications	Advantages	Disadvantages/limitations
Active Surveillance (Observation)	Small, incidental, asymptomatic lesions; Low Cyst Index (Humerus <4, Femur <3.5).	Avoids surgical risks; Utilizes natural potential for spontaneous regression.	No curative effect for high-risk lesions; Requires frequent, long-term follow-up.
Steroid Injection	Traditional option for active cysts; Families preferring outpatient care.	Minimally invasive; Low cost; Technically simple.	High recurrence; Often requires multiple sessions (lowered success rate in recent studies).
BM & DBM Injection	Active cysts where biological regeneration is prioritized over mechanical support.	High osteogenic potential; Uses autologous stem cells and inductive scaffolds.	Variable outcomes (23%–76% healing); Requires bone marrow aspiration
Bone Substitute Injection	Lesions requiring rapid density recovery; Fracture-prone cysts in non-weight-bearing bones.	Provides immediate structural density; Superior biocompatibility and safety.	Mismatch between resorption and new bone formation; Higher material costs.

Steroid injection remains one of the earliest methods employed for treating UBCs in children. The relative simplicity of the procedure, its minimal adverse effects, and the ability to perform it in an outpatient setting established corticosteroid injections, particularly with methylprednisolone acetate (MPA), as a widely adopted clinical approach. The mechanism is thought to involve the microcrystals of the corticoid dissolving and disrupting the connective tissue of the cyst wall. This action is believed to promote the ingrowth of adjacent capillaries into the cavity, thereby facilitating the cyst's ultimate consolidation. The method gained prominence following the work of Scaglietti et al. in 1979, who introduced the intraosseous injection of MPA and recommended repeating the treatment until the cyst healed (19). Initial reports suggested high success rates: as early as 1982, R. Capanna (20) reported satisfactory results in 80% of 95 UBCs patients treated with MPA, and a later long-term follow-up showed an overall good response in 82.6% of patients (21).

However, the efficacy of steroid injection has been inconsistently demonstrated in more recent literature. While Scaglietti initially described healing rates up to 90%, subsequent studies have reported lower success rates, often necessitating multiple injections. For instance, more contemporary studies indicate resolution rates between 33% and 41% (22). Similarly, Bella's (23) study reported a healing rate of only 38% in the group treated with multiple steroid injections. The varied and often suboptimal outcomes were further highlighted in a randomized clinical trial by Wright (24), where only 16 of 38 cysts (42%) treated with MPA healed. Due to the morbidity associated with multiple injections and the persistent risk of pathological fractures while awaiting healing, the historical treatment of intraosseous MPA injection is currently less frequently used and is no longer uniformly recommended as a first-line therapy.

#### Bone marrow and demineralized bone matrix

The percutaneous injection of autologous bone marrow (BM) combined with demineralized bone matrix (DBM) constitutes a regenerative therapeutic strategy for simple bone cysts (SBCs) in pediatric patients. This approach leverages the synergistic roles of its components: BM serves as a source of mesenchymal stem cells (MSCs), which are capable of osteogenic differentiation and secrete immunomodulatory cytokines critical for bone repair; concurrently, DBM provides an osteoconductive scaffold that delivers inherent bone morphogenetic proteins (BMPs), thereby enhancing osteo-induction and offering mechanical support during remodeling (25–27).

Evidence regarding the efficacy of this technique, however, remains heterogeneous. While initial studies by Lokiec et al. (28) reported complete success with autologous BM monotherapy, subsequent investigations have yielded variable outcomes. Later research supports the combination therapy, with studies such as that by Rougraff and Kling demonstrating the effectiveness of allogeneic DBM plus autogenous BM (27). One follow-up study (>2 years) documented a 76% healing rate (defined as >50% reduction in cyst volume) in 21 active cysts after up to three injections of DBM and autogenous BM (29). Conversely, a randomized trial by Wright (30) reported a considerably lower success rate, with only 23% (9/39) of cysts healing after treatment with bone marrow alone, highlighting the inconsistency in clinical outcomes compared to other minimally invasive options like steroid injections.

#### Bone graft substitutes

Traditional surgical treatments for unicameral bone cysts (UBCs), including autogenous, allogeneic, and xenogeneic bone grafts, remain standard but carry significant drawbacks. These complications include donor site morbidity, the risk of immune rejection, and variable integration rates (31). Consequently, synthetic and bioengineered bone substitutes have become a central focus in regenerative medicine. The use of these artificial materials offers distinct advantages by eliminating the trauma associated with autograft harvesting, thereby reducing operative and hospitalization times. Recent advancements have introduced diverse new types of substitutes, such as novel synthetic bone grafts and modified biomaterials, engineered to more closely mimic the structure and function of natural bone and enhance cyst healing.

Calcium sulfate, one of the earliest synthetic bone graft substitutes, is widely used in orthopedic and dental surgery due to its excellent biocompatibility and complete *in vivo* resorption. However, its main drawback is a mismatch between its rapid resorption rate and the slower pace of new bone formation, potentially leading to incomplete defect regeneration unless combined with other materials (32). In a retrospective study, 13 pediatric UBC patients received a single percutaneous calcium phosphate injection. Healing outcomes included complete healing in 5 cysts, substantial healing (>50%) in 6, partial healing with cortical thickening in 2, and no recurrence. These findings support this method as a safe, minimally invasive, and effective treatment (33). Clinical studies have supported the efficacy of various substitutes: In a 38-patient series, bioresorbable bone cements provided stability and effectively prevented refracture in UBCs located in the upper extremity or foot. For proximal femoral cysts, however, additional stabilization is required due to weight-bearing demands and a higher associated refracture rate (34). In a retrospective study, percutaneous calcium sulfate injection offers a superior minimally invasive option for pediatric humeral pathologic fractures, with advantages including reduced operative time, bleeding, and postoperative pain compared to bone grafting (35). Xu et al. (36) reported successful bone healing in 32 of 36 pediatric patients treated with percutaneous intramedullary aspiration and injection of absorbable bone substitutes, highlighting the treatment's safety and effectiveness. Their study utilized two types of absorbable graft materials: calcium sulfate/calcium phosphate matrix mixed with beta-tricalcium phosphate, and bioactive calcium phosphosilicate. Our own retrospective review further contributes to this evidence, demonstrating that bone substitute injection (specifically a combination of calcium sulfate and calcium phosphate) is a potentially successful, minimally invasive treatment for UBCs, achieving an 80% radiographic resolution rate in 15 patients (37).

### Surgical treatment

The management of UBCs through surgical intervention is guided by the primary objectives of preventing pathologic fractures, restoring cortical integrity, and minimizing the risk of recurrence. While the treatment spectrum ranges from observation to extensive open procedures, no definitive consensus exists regarding the optimal surgical technique, the timing of intervention, or uniform indications for surgery. Despite this, expert opinion, as highlighted by a joint survey of EPOS and POSNA members, generally recommends surgery for painless UBCs that carry a high risk of fracture (17). To bring structure to this complex decision-making landscape, Paez et al. proposed a decision tree algorithm. Although adherence to this algorithm did not yield a statistically significant increase, it was associated with a numerically higher healing rate (75% vs. 67%), underscoring its practical value in standardizing a stepwise and minimally invasive treatment pathway (38). Various treatment modalities, including simple curettage, decompression with elastic stable intramedullary nailing, and the use of bone graft substitutes, have been reported, though a best-practice approach has yet to be determined (Table 2).

**Table 2:** Surgical management strategies for UBCs.

Treatment modality	Indications	Advantages	Disadvantages/limitations
Simple Curettage	Large, symptomatic, or recurrent cysts; Failure of conservative or injection methods.	Direct elimination of cyst lining;	More invasive; Variable success rates.
ESIN & Decompression	Active cysts in long bones (humerus/femur) with high fracture risk.	Triple Action: Internal fixation, continuous pressure decompression, and osteogenesis.	Potential hardware irritation or secondary surgery for nail removal.
Curettage + ESIN + Grafting	High-risk or recurrent lesions in weight-bearing bones (e.g., proximal femur).	Combines stability (ESIN), biological clearing (Curettage), and scaffold support. Highest success rate.	Longer operative time; Technically demanding; Increased surgical trauma.
Endoscopic Surgery	Calcaneal or talar UBCs; Athletes requiring rapid return to activity.	Excellent visualization; Minimal soft tissue and cartilage damage; Extremely fast recovery.	Requires specialized endoscopic equipment and high technical proficiency.

#### Simple curettage

Curettage remains a classic and widely utilized surgical approach, particularly indicated for cysts that are active, at high risk of pathologic fracture, or those that have failed conservative management. The fundamental objective of this procedure is to eliminate the cyst's biological activity by thoroughly debriding the lining and internal contents, thereby reducing the risk of recurrence. This process is frequently augmented by various adjuvant therapies—such as high-speed burring, high-frequency electrocautery, cryotherapy (liquid nitrogen), or phenol application—which are employed to enhance local control rates. Following curettage and local inactivation, the resulting osseous defect necessitates filling with autograft, allograft, or synthetic bone substitutes to provide structural support, promote bone healing, and restore mechanical strength.

However, the technique has notable drawbacks, including its relatively invasive nature and the requirement for bone defect reconstruction. Furthermore, the literature reports a variable success rate, with considerable disagreement depending on the specific cyst type and the material used for defect filling. For instance, reported success rates for curettage range from 53% to 78% (39–41). In a retrospective study of 23 patients found that while most open procedures for UBCs had failure rates comparable to minimally invasive techniques, the specific subgroup treated with curettage, allograft, and adjuvants exhibited a significantly higher failure rate, with a five-year failure-free survival of only 50.4% (42). Celik reported a recurrence rate of 18.75% (3 out of 16 patients) in a study of unicameral bone cysts (41). In the context of unicameral bone cysts (UBCs), Sung et al. reported a high failure rate of 64% (25 of 39 patients) treated with curettage and bone grafting (40). A systematic review of 4,973 UBCs demonstrated that the success rate of curettage followed by grafting—using autograft, allograft, or bone substitutes—varied from 69% to 88%, depending on the specific filling material employed (43).

#### Elastic stable intramedullary nail and decompression

The adoption of minimally invasive techniques is driven by their capacity to significantly reduce postoperative morbidity, accelerate recovery time, and minimize both cosmetic and functional impairments. By utilizing smaller incisions, these procedures reduce iatrogenic injury to surrounding tissues, thereby enhancing the patient's quality of life and potentially lessening the psychological impact of surgery, offering a safer and more effective treatment option (35). ESIN stems from its tripartite mechanism of action: it provides internal fixation for immediate structural support; it facilitates decompression and drainage, lowering intracystic pressure to purge inhibitory agents such as prostaglandin E2 and lysosomal enzymes; and finally, it stimulates osteogenesis to actively promote surrounding new bone formation.

Given these advantages, there has been increasing enthusiasm for simple cyst decompression without mandatory grafting (e.g., percutaneous curettage and decompression using flexible intramedullary nails) (44). Indeed, some literature suggests that decompression of the cyst wall is more critical for improving the healing rate than the type of graft used to fill the cavity (14). The application of ESIN not only lowers the risk of postoperative complications but also facilitates early restoration of patient mobility. For instance, a controlled study on 50 patients with humeral bone cysts treated with ESIN drainage reported a 50% recurrence rate, with an average of 2.8 procedures required to achieve healing (45). A multicentre retrospective study across 20 paediatric hospitals in France, Belgium, and Switzerland (1995–2017) on proximal femoral UBCs found that ESIN insertion—alone or combined with percutaneous injection or curettage and grafting—may achieve higher healing rates than other surgical procedures (1). Furthermore, the relative simplicity of the ESIN technique makes it a practical and advantageous choice for use in the pediatric population. Due to its favorable outcomes in UBCs healing and low complication rates, continuous decompression of the cyst cavity using elastic intramedullary nails has garnered increased attention in recent years (43, 46).

#### Curettage with grafting and internal fixation

For larger, high-risk, or recurrent Unicameral Bone Cysts (UBCs), curettage of the cystic contents is typically supplemented by bone grafting. The goals of this combined approach are twofold: surgically remove the pathological tissue and introduce an osteoconductive scaffold to promote bone regeneration and healing. Specifically, curettage and bone grafting serve to fill the post-curettage osseous defect, restore structural integrity to prevent fracture, and contain cyst recurrence. This strategy is often viewed alongside the finding that continuous decompression, rather than the specific choice of graft material, may be more critical for enhancing the healing rate (14). Literature suggests that the addition of grafting significantly improves bone healing and reduces recurrence compared to simple curettage alone (47). In a retrospective study of 83 cases, treatment with autologous bone marrow injection combined with ESIN demonstrated the highest effectiveness and cure rates for pediatric bone cysts (48). A meta-analysis found that curettage and bone grafting combined with elastic intramedullary nailing is superior to curettage and bone grafting alone for treating simple bone cysts, with a lower incidence of postoperative refracture (49).

Moreover, the integration of ESIN and curettage has yielded excellent results. The “three-in-one” procedure, which combines ESIN, curettage, and calcium phosphate cement application, was evaluated in a retrospective study of 116 children presenting with pathological fractures secondary to large benign bone lesions. This combined protocol proved highly efficient and successful, reporting a 100% healing rate and no recurrences during a mean two-year follow-up period (50). Supporting this multimodal approach, a systematic review of 4,973 UBCs found that the highest success rates were associated with curettage plus ESIN combined with bone graft or substitute for defect packing. Specifically, while the success rate of ESIN without curettage was 87.91%, performing curettage prior to ESIN placement increased the success rate to 91.16% (43).

#### Endoscopic surgery

Minimally invasive endoscopic surgery has emerged as an increasingly favored approach for managing UBCs, by offering enhanced intraoperative visualization, reduced invasiveness, and shorter immobilization periods. This approach demonstrates excellent safety and efficacy in the pediatric population, facilitating a significantly accelerated postoperative recovery and allowing patients to resume normal activities rapidly with a low recurrence rate, thus representing an increasingly desirable treatment choice. For instance, one case report documented the successful use of an endoscopically assisted curettage and allograft technique for a symptomatic calcaneal UBCs, confirming its adequacy (51).

A dedicated study on young athletes with symptomatic calcaneal UBCs found that endoscopic curettage combined with bone substitute injection was an excellent option. The procedure yielded significant clinical improvement and enabled a rapid return to sports (mean 7.1 weeks) without reported recurrence or fracture (52). Furthermore, a retrospective case series (*N* = 37) confirmed that endoscopic curettage for simple bone cysts is a minimally invasive and effective alternative, reporting a 18.9% recurrence rate and achieving solid bone union in an average of 4.0 months, though they noted a correlation between physis contact and recurrence (53). Similarly, an arthroscopically assisted anterior treatment with bone grafting was successfully applied to seven cases of symptomatic large talar bone cysts. This technique effectively overcame the limitations of traditional open surgery by allowing lesion removal, ablation, and filling while minimizing talar cartilage damage. The procedure resulted in a statistically significant improvement in function (mean AOFAS score increased from 65 to 91, *P* < .01) and exhibited no recurrence or complications after one year (54). Given the continuously evolving nature of endoscopic technology and its demonstrated success and safety profile, its application in pediatric UBCs treatment holds considerable promise and is poised to potentially become a standard treatment methodology in the future.

## Conclusion and author suggestion

Considering the natural history of pediatric UBCs, which are generally regarded as a self-limiting benign condition, the primary indication for active treatment is the potential risk of pathologic fracture. A high recurrence rate has been correlated with an elevated cyst index, which in turn corresponds to a higher fracture risk.

**Figure 2:**
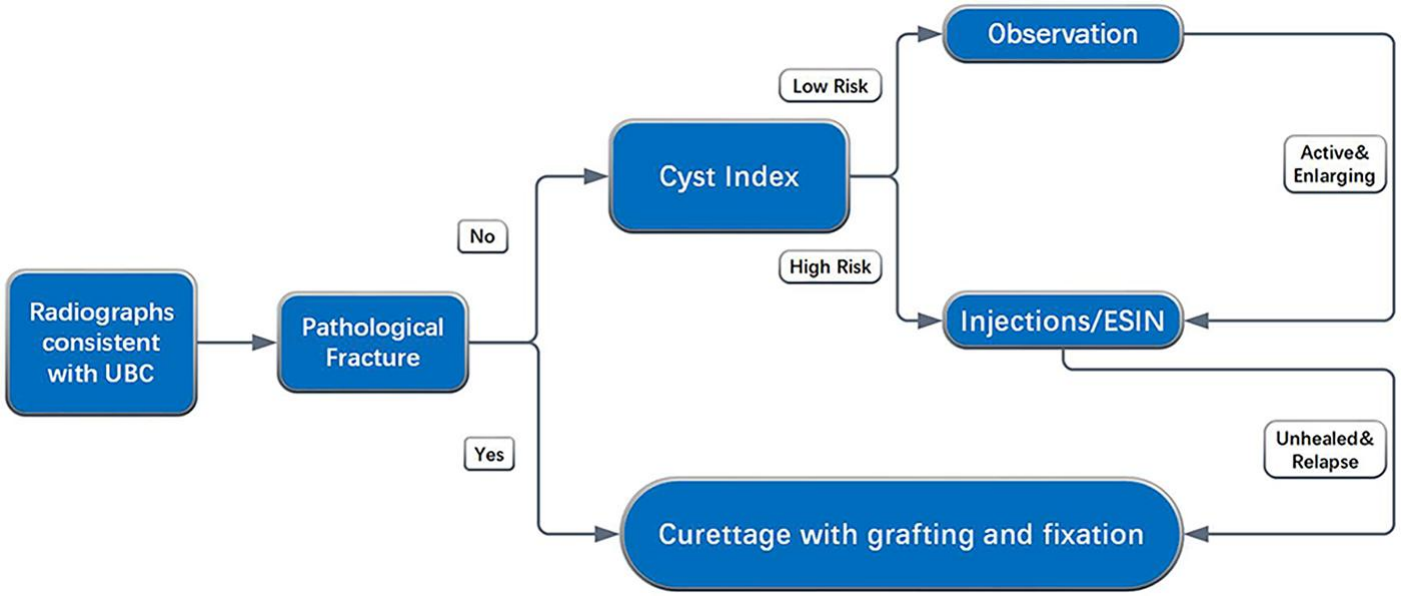
UBCs treatment suggestion.

Following radiographic diagnosis, specific treatment pathways are recommended (Figure 2).

1. For patients presenting with a pathologic fracture, treatment should involve curettage, bone grafting, and internal fixation to promote healing and provide stability.
2. For patients without fracture, the cyst index should be used to stratify the risk of future fracture. High-risk patients are ideally treated with minimally invasive methods, such as injection therapy or minimally invasive Elastic Stable Intramedullary Nailing (ESIN), to facilitate osseous consolidation.
3. Low-risk patients are managed with conservative treatment and regular follow-up. During this period, the limb must be protected, and activity restricted to minimize fracture risk and prevent subsequent pathologic events.
