# Correction: Management strategies and outcomes of pediatric unicameral bone cyst: a narrative review

**DOI:** 10.3389/fped.2026.1839679

**Published:** 2026-04-29

**Authors:** Frontiers Production Office

**Affiliations:** Frontiers Media SA, Lausanne, Switzerland

**Keywords:** bone cyst, curettage, pathological fracture, pediatric, treatment

An incorrect Acceptance date (31 December 2025) was given for this article on publication. The correct Acceptance date is 17 February 2026.

The original version of this article has been updated.

